# Sea Hare Hydrolysate Reduces PD‐L1 Levels in Cancer Cells and Mitigates Rheumatoid Arthritis Ina Collagen‐Induced Arthritis Mouse Model

**DOI:** 10.1002/fsn3.4644

**Published:** 2024-12-02

**Authors:** Ji Hyeon Ryu, Min Seok Song, Marie Merci Nyiramana, Anjas Happy Prayoga, Dang Long Cao, Gyeong‐Won Lee, Hyuk‐Kwon Kwon, Dawon Kang

**Affiliations:** ^1^ Research Institute for Convergence of Biomedical Science and Technology Pusan National University Yangsan Hospital Yangsan Republic of Korea; ^2^ Department of Physiology, College of Medicine Gyeongsang National University Jinju Republic of Korea; ^3^ Institute of Medical Sciences Gyeongsang National University Jinju Republic of Korea; ^4^ Department of Convergence Medical Science Gyeongsang National University Jinju Republic of Korea; ^5^ Department of Internal Medicine, College of Medicine Gyeongsang National University Jinju Republic of Korea; ^6^ Division of Life Science Gyeongsang National University Jinju Republic of Korea

**Keywords:** arthritis, cancer, immunotherapy, PD‐L1, sea hare hydrolysate

## Abstract

Our previous study highlighted the anticancer potential of sea hare hydrolysate (SHH), particularly its role in regulating macrophage polarization and inducing pyroptotic death in lung cancer cells through the inhibition of signal transducer and activator of transcription 3 (STAT3). These findings prompted us to investigate additional features of immune‐oncology (I‐O) agents or adjuvants, such as programmed cell death protein 1 (PD‐1)/programmed death ligand 1 (PD‐L1) inhibition and their association with rheumatoid arthritis (RA) risk, to explore the potential of SHH as an I‐O agent or adjuvant. In this study, we investigated the effects of SHH on PD‐L1 levels in various cancer cell types and assessed its effectiveness in treating RA, a common side effect of I‐O agents. Our results showed a marked reduction in PD‐L1 levels in multiple cancer cell lines and decreased PD‐1 and PD‐L1 levels in tumor‐associated macrophages. In a mouse model with collagen‐induced arthritis (CIA), SHH exhibited anti‐inflammatory effects comparable to methotrexate (MTX), a first‐line treatment for RA. Both the SHH and MTX groups had significantly lower arthritis scores and paw thickness compared to the CIA group. Additionally, SHH or MTX treatment effectively reduced elevated levels of anticollagen type II (CII) antibodies and proinflammatory cytokines (IL‐1β, IL‐6, and TNF‐α). Histopathological analysis revealed that SHH and MTX treatments notably mitigated arthritic inflammation, synovial hyperplasia, and loss of articular cartilage and bone. Micro‐CT scans showed reduced articular destruction in the SHH and MTX groups. These findings indicate that SHH treatment decreases PD‐L1 levels in cancer cells and reduces the severity of CIA by exerting anti‐inflammatory effects. Therefore, SHH holds promise as an I‐O agent without side effects such as exacerbation of RA.

## Graphical abstract

SHH reduced PD‐L1 levels in multiple cancer cell types. SHH exhibited anti‐inflammatory effects in a collagen‐induced arthritis mouse model. SHH holds promise as an immune‐oncology agent with a lower risk of exacerbating RA.

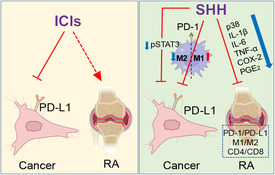

## 1 Introduction

Immune checkpoint inhibitors (ICIs), a key class of immune‐oncology agents, include antibodies targeting programmed cell death protein 1 (PD‐1) and programmed cell death ligand 1 (PD‐L1). These agents are revolutionizing cancer therapy due to their broad applicability and durable clinical outcomes. However, the use of these therapies is not without challenges, as they can trigger autoimmune responses and, in some cases, promote unintended tumor growth (He and Xu 2020). ICIs are known to frequently cause immune‐related adverse events (irAEs), complicating the treatment of conditions like arthritis‐irAEs. Therefore, administering ICIs to cancer patients with arthritis necessitates careful management of these adverse effects to preserve the antitumor efficacy of the treatment (Kim et al. 1970). Consequently, it is essential to grasp the mechanisms behind irAEs thoroughly and to develop anti‐inflammatory treatments and adjuvants that effectively control these events without diminishing the cancer‐fighting immune response. Moreover, a promising approach involves therapies that adjust PD‐L1 levels on tumor cells, potentially enhancing the effectiveness of ICIs.

The overall cancer risk in patients with rheumatoid arthritis (RA) is approximately 20% higher than in the general population, with a significant increase in cancers commonly associated with tobacco use, such as lung, bladder, and head and neck cancers, as well as cervical, prostate, melanoma, and hematological malignancies. Notably, patients receiving biotherapies like rituximab or abatacept, which target B‐cell depletion or T‐cell inhibition, are at the highest risk of developing cancer (Beydon et al. 2023; Kalliolias, Basdra, and Papavassiliou 2023). The PD‐1 and PD‐L1 pathway plays a crucial role in maintaining immune tolerance and preventing excessive immune responses. While this pathway is primarily recognized for its role in regulating immune responses, its dysregulation in RA may contribute to the chronic inflammation and immune overactivation observed in the disease. Studies have shown that PD‐1 and PD‐L1 expression is upregulated in RA synovial tissues (the lining of the joints), suggesting that the PD‐1/PD‐L1 axis is involved in the pathogenesis of RA by modulating T‐cell responses and promoting inflammation (Canavan et al. 2021; Raptopoulou et al. 2010; Luo et al. 2018).

Sea hare hydrolysate (SHH), derived from marin mollusks of *Aplysia kurodai* (*A. kurodai*), has exhibited various pharmacological activities, including anti‐inflammatory, antioxidant, and anticancer effects, due to its bioactive compounds (Ryu et al. 2017; Nyiramana et al. 2020). Recent findings highlight SHH's anticancer effects on non‐small‐cell lung cancer cells (NSCLC), notably through its ability to activate M1 macrophages, reduce M2 macrophage function, and inhibit tumor growth by blocking STAT3 activity (Nyiramana et al. 2020). These mechanisms suggest SHH's potential role in cancer immunotherapy. Furthermore, SHH's anti‐inflammatory properties, as demonstrated in allergic bronchial asthma models (Ryu et al. 2017), resemble immune and inflammatory reactions seen in RA. Prompted by these results, our research was directed towards determining if SHH affects PD‐L1 expression in cancer cells and its effectiveness in reducing RA symptoms.

## 2 Materials and Methods

### 2.1 Producing Sea Hare Hydrolysates

Creating SHH was conducted following the method outlined in a previous study (Nyiramana et al. 2020). The sea hare species *A. kurodai* was subjected to extensive cleaning, blanching, and fine chopping. The chopped sea hare was then treated with a 2% flavourzyme solution. The subsequent incubation and extraction steps were carried out as previously described (Nyiramana et al. 2020). The resulting powdered product was reconstituted to the experiment's required concentrations in distilled water.

### 2.2 Culture of Cancer Cell Lines

Multiple human cancer cell lines were employed, including breast cancer lines MCF‐7 and MDA‐MB‐231, NSCLC lines A549 and HCC‐366, the colorectal cancer line SW620, the kidney cancer line A498, and ovarian cancer lines NCI/ADR‐RES (ART‐CRL‐3468) and SK‐OV‐3. The ovarian cancer lines were obtained from the American Type Culture Collection (Manassas, VA, USA). The remaining cell lines were procured from the Korean Cell Line Bank (KCLB, Seoul, Korea). These cell lines were cultured in either Dulbecco's modified Eagle's medium (DMEM; Gibco‐BRL, Gaithersburg, MD, USA) or a DMEM/F12 blend (for SW620 and SK‐OV‐3), supplemented with 10% fetal bovine serum (FBS; Gibco‐BRL) and 1% penicillin/streptomycin (100 μg/mL). Cultivation occurred under a 5% CO_2_ environment at 37°C, with medium replacements every 2 days.

### 2.3 Production of Tumor‐Associated Macrophages (TAMs)

TAM production followed the previous study's methods (Nyiramana et al. 2020). The human monocytic cell line THP‐1, obtained from the KCLB, was used to produce TAMs. These THP‐1 cells, with a 2 × 10^5^ cells/mL density, were seeded into a 6‐well plate and cultured for 24 h in RPMI medium (Lonza, Walkersville, MD, USA). Phorbol myristate acetate (PMA, 150 nM, Sigma‐Aldrich, St. Louis, MO, USA) was introduced to prompt the transformation of cells into macrophage‐like cells. After 24 h, these differentiated macrophages were subsequently co‐cultured with A549 cells in equal proportions for 4 days to support the development of TAMs.

### 2.4 Reverse Transcriptase (RT)‐polymerase Chain Reaction (PCR)

RT‐PCR followed the previous study's methods (Nyiramana et al. 2020). The PCR amplification was conducted using designated primers targeting the genes of interest (Table 1). The PCR protocol began with an initial denaturation at 94°C for 5 min, followed by 32 cycles consisting of denaturation at 94°C for 30 s, annealing at 55°C to 60°C for 30 s, and extension at 72°C for 30 s. The process concluded with a final extension at 72°C for 10 min. Following PCR, the products were run through a 1.5% agarose gel, stained with ethidium bromide, and the specific bands were excised for sequencing using an ABI PRISM 3100‐Avant Genetic Analyzer (Applied Biosystems, Carlsbad, CA, USA).

**TABLE 1:** Primer sequences used for RT‐ PCR.

Gene name	GenBank acc. no.	Primer sequences (5′–3′)	Expected size (bp)
*PD*‐1	L27440.1	Sense: TATGGTGGTGCCGACTACAA Antisense: CCCATAGTCCACAGAGAACACA	492
*PD*L1	AY254342.1	Sense: TATGGTGGTGCCGACTACAA Antisense: TGGCTCCCAGAATTACCAAG	388
*GAPDH*	NM_002046.7	Sense: CCCATGTTCGTCATGGGTGT Antisense: TGGTCATGAGTCCTTCCACGATA	145

### 2.5 Animal Care

Male DBA/1 J mice, 7 to 8 weeks old, were purchased from Central Lab. Animal Inc. (Seoul, Korea). Maintained in a sterile environment, these mice had free access to food and water and were housed at a stable temperature of 22°C ± 2°C. The mice's body weights were recorded at the outset on the surgery day and every week. All experimental protocols were approved by the Institutional Animal Care and Use Committee of Pusan National University, adhering to the National Institutes of Health Guidelines (PNU‐2020‐2757).

### 2.6 Establishment of the Collagen‐Induced Arthritis (CIA) Mouse Model and Experimental Group Setup

CIA induction in mice was performed using well‐established protocols (Canavan et al. 2021). Initially, on day 0, mice received an intradermal injection at the base of their tails with 100 μg of bovine type II collagen (CII; Chondrex Inc., Redmond, WA, USA), combined in equal parts with complete Freund's adjuvant (CFA; Sigma‐Aldrich). A booster immunization was administered on day 21, consisting of a mixture of CII and incomplete Freund's adjuvant (Chondrex). In parallel, control mice were given PBS injections concurrently with the CII immunizations. Postbooster, the mice were allocated into four distinct groups (each containing 7–8 mice): a sham group, a CIA group, a CIA group treated with SHH at a dosage of 1 g/kg/day, and a CIA group receiving Methotrexate (MTX; Sigma‐Aldrich) dosed at 1 mg/kg every 3 days. The SHH treatment was administered orally from day 22 to day 35. Control mice received oral doses of distilled water. MTX, frequently utilized in rheumatoid arthritis treatment, was injected intraperitoneally into the assigned group every 3 days from day 22 through day 35.

### 2.7 Clinical Assessment Protocol

The mice were subjected to a comprehensive monitoring schedule postcollagen injection, with evaluations occurring thrice weekly. Each evaluation involved the independent examination of each limb by three separate investigators. Based on the criteria, a 4‐point scoring system was used (Brand, Latham, and Rosloniec 2007). The overall arthritis score was calculated by adding the scores from all four paws.

### 2.8 Histological Analysis

Tissue staining of ankle joints adhered to methods outlined in a previous study (Ryu et al. 2023). The ankle joints were excised on day 36 of the experimental period. Synovial inflammation and hyperplasia were analyzed through H&E staining, while cartilage damage was evaluated with safranin‐O staining using a four‐point criteria system. The stained sections were examined using a virtual microscope (Axio Scan.Z1; Carl Zeiss, Heidenheim, Germany). Three blinded observers independently conducted these histological evaluations; the results presented are the mean scores from these assessments.

### 2.9 Microcomputed Tomography (CT) Imaging

Micro‐CT imaging was performed using previously established methods (Ryu et al. 2023). On day 36, the hind paws of mice from each group were scanned using a Quantum FX micro‐CT scanner (Perkin Elmer, MA, USA). Ankle joints were scanned with a tube voltage of 90 kV and a tube current of 160 μA. The resolution of the scans was maintained at 20 μm, and each scan was timed to last 2 min.

### 2.10 Enzyme‐Linked Immunosorbent Assay (ELISA)

Blood samples were obtained from the mice through cardiac puncture 24 h following the final treatment. The samples were then centrifuged to separate the serum. The concentrations of mouse anti‐CII antibodies (IgG, IgG1, and IgG2a) were determined using ELISA kits (Chondrex). Additionally, levels of mouse tumor necrosis factor (TNF)‐α, interleukin (IL)‐1β/12, and IL‐6 were assessed using ELISA kits provided by R&D Systems (Minneapolis, MN, USA), according to the manufacturer's guidelines. Absorbance readings were taken at 450 nm using a microplate reader (Tecan Infinite M200 PRO, Männedorf, Switzerland).

### 2.11 Assessment of Liver Safety

Blood samples were obtained 24 h postfinal treatment for liver function assessment. Serum concentrations of alanine transaminase (ALT) and aspartate transaminase (AST) were measured. GC Labs (Yongin, Gyeonggi, Korea) conducted these analyses following the International Federation of Clinical Chemistry standard method, in line with protocols detailed in prior research (Siregar et al. 2020).

### 2.12 Statistics

Data are displayed as mean ± standard deviation (SD). Either one‐way ANOVA with Bonferroni's post hoc test or the Kruskal‐Wallis test augmented by the Mann–Whitney test was employed to assess group differences. The appropriate test selection was based on the results of a normality assessment conducted using OriginPro2020 (OriginLab Corp., Northampton, MA, USA). A *p*‐value of less than 0.05 was considered statistically significant.

## 3 Results

### 3.1 Reduction of PD‐L1 Expression in Cancer Cells by SHH

**FIGURE 1:**
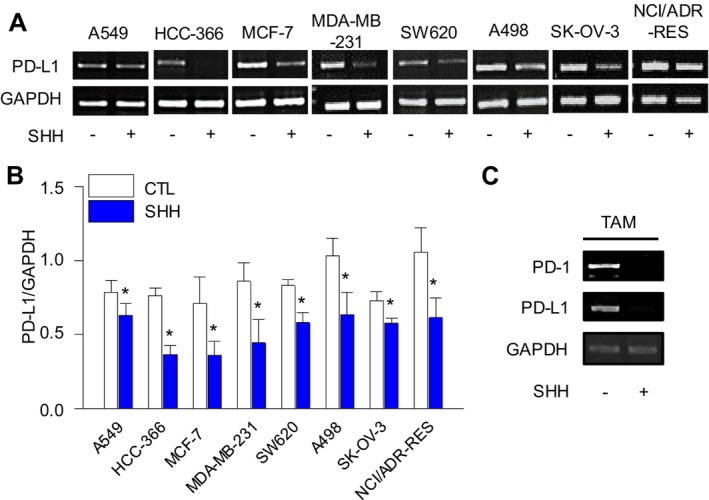
Reduction of programmed death ligand 1 (PD‐L1) expression in various cancer cells and tumor‐associated macrophages (TAM) after treatment with SHH. (A and B) Significant reduction in the expression level of PD‐L1 in different cancer cells after treatment with SHH. (C) Decrease in PD‐1 and PD‐L1 expression in TAM with SHH treatment. Data are presented as mean ± SD of four independent experiments. The + and − marks indicate treatment and nontreatment of SHH. Significance was considered at *p* < 0.05. *p < 0.05 compared to each corresponding control.

SHH (100 μg/mL) treatment significantly reduced PD‐L1 expression in several cancer cell lines: NSCLC (A549 and HCC‐366), breast cancer (MCF‐7 and MDA‐MB‐231), colorectal cancer (SW620), kidney cancer (A498), and ovarian cancer (SK‐OV‐3 and NCI/ADR‐RES) (Figure 1A,B, *p* < 0.05). In addition, a notable decrease in PD‐1 and PD‐L1 expression was observed in TAMs (Figure 1C).

### 3.2 Anti‐Inflammatory Effect of SHH in a Mouse Model of Collagen‐Induced Arthritis

**FIGURE 2:**
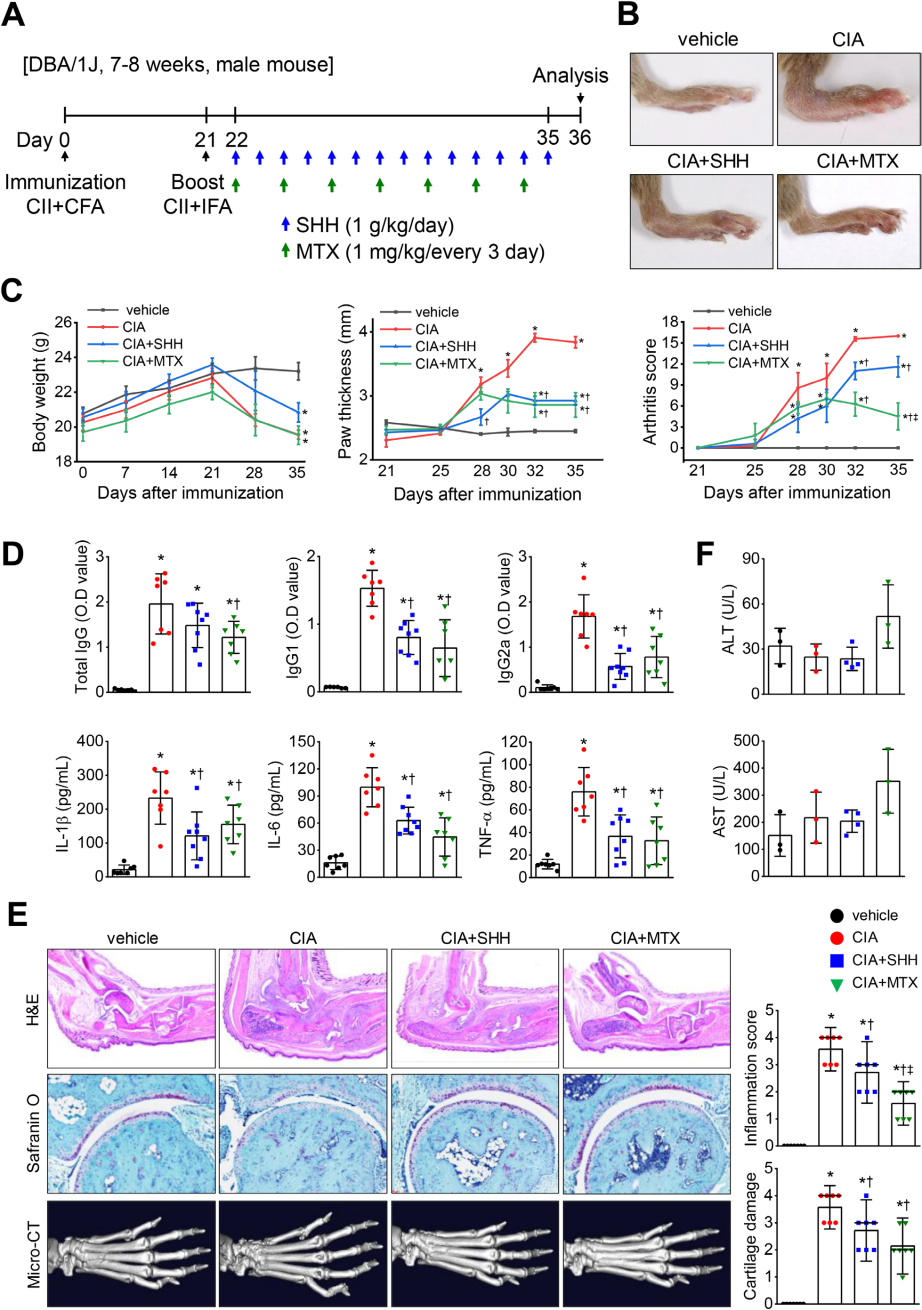
Anti‐inflammatory effect of SHH on collagen‐induced arthritis (CIA) mouse model. (A) Experimental protocol for CIA mouse model and intervention. (B and C) Reduced inflammatory responses in the paws of CIA mice treated with SHH, reflecting lower arthritis scores and paw thickness. (D) Decreased levels of anticollagen type II (CII) immunoglobulins and proinflammatory cytokines in serum after SHH treatment. (E) Histopathological analysis of ankle joint in CIA mice. Notable mitigation of arthritic inflammation and loss of articular cartilage and bone in CIA mice post SHH treatment. (F) NO hepatotoxic effects of long‐term SHH administration. Data are shown as mean ± SD. Seven mice were used in each group, with eight in the SHH group. Blood from three mice per group was used for liver enzyme assays. *p < 0.05 compared to sham group. †*p* < 0.05 compared to the CIA group. ‡*p* < 0.05 compared to CIA + SHH group.

The effect of SHH on inflammatory arthritis was assessed using a well‐established collagen‐induced arthritis (CIA) mouse model, comparing SHH treatment with the standard rheumatoid arthritis (RA) drug, methotrexate (MTX). The experimental timeline is detailed in Figure 2A. As shown in Figure 2B,C, CIA mice treated with SHH exhibited lessened inflammation in the paws than in the CIA group. Both SHH and MTX treatments yielded significantly reduced arthritis scores and paw thickness. Furthermore, while the CIA group had elevated anticollagen type II (CII) antibodies, such as total immunoglobulin G (IgG), IgG1, and IgG2a, these were notably decreased by either SHH or MTX treatment (Figure 2D upper panel). Elevated pro‐inflammatory cytokine (IL‐1β, IL‐6, and TNF‐α) in the CIA mice serum were substantially reduced post‐SHH or ‐MTX administration (Figure 2D bottom panel). Histological analysis revealed that the CIA mice exhibited arthritic inflammation, synovial hyperplasia, and articular cartilage and bone degradation. Both treatments considerably alleviated these conditions in the ankle joint (Figure 2E). Micro‐CT scans further corroborated a decline in joint damage in the SHH and MTX groups (Figure 2E bottom). Long‐term administration of SHH and MTX demonstrated no significant liver toxicity, with ALT and AST levels comparable to the vehicle group (Figure 2F).

## 4 Discussion

This study builds on our previous work and reinforces the potential of SHH as an I‐O agent. In addition, SHH offers the advantage of not exacerbating RA; instead, it alleviates RA symptoms through its anti‐inflammatory effects. We found that SHH reduced the expression of PD‐L1 in several cancer cells. PD‐L1, expressed in specific cancer cells, interacts with T‐cells, enabling cancer cells to evade immune system attacks. Therefore, the reduction of PD‐L1 expression by SHH could potentially increase the vulnerability of these cells to immune cell targeting and subsequent attacks. High levels of PD‐L1 in cancer cells enhance their growth, migration, and invasion while reducing PD‐L1 levels inhibits these processes (Yu et al. 2020; Yoon et al. 2022). Additionally, one of the key mechanisms behind cancer immune escape is the loss of specific miRNAs that normally suppress PD‐L1 expression in tumor cells (Wang et al. 2017).

The tumor microenvironment (TME), comprising diverse immune cells, is pivotal in cancer cell tumorigenesis, proliferation, and metastasis. TAMs are predominantly distributed within these immune cells (Zhang et al. 2023). They contribute to forming a tumor‐promoting TME by modulating PD‐L1 expression on cancer cells and releasing various cytokines. However, SHH can counteract this by diminishing the levels of PD‐1 and PD‐L1 on TAMs, fostering a tumor‐suppressive TME. Anti‐PD‐L1 interventions elevate markers linked with M1‐like macrophages and decrease those related to M2‐like macrophages (Zhang et al. 2023; Xiong et al. 2019), similar to the effect exerted by SHH (Nyiramana et al. 2020). M2 macrophages exhibited a higher expression of PD‐1 compared to the M1 macrophages (Xu et al. 2023). TAMs expressing PD‐1 exhibit lower phagocytic abilities than those lacking PD‐1 (Gordon et al. 2017). In addition, PD‐1 plays a role in inducing macrophage apoptosis, mediated by inhibiting the AKT pathway (Roy et al. 2017). Reduction of PD‐1 expression by SHH might lead to diminished macrophage apoptosis, potentially boosting their phagocytic ability.

In the CIA mouse model, compared to MTX, SHH demonstrated similar anti‐inflammatory and antiarthritis effects. MTX exerts its anti‐inflammatory effects through various mechanisms, including inhibiting purine and pyrimidine synthesis, influencing NF‐κB activation, modulating JAK–STAT signaling, controlling nitric oxide (NO) production, and promoting adenosine release (Cronstein and Aune 2020). The anti‐inflammatory mechanisms of SHH overlap with some of those of MTX. SHH shows anti‐inflammatory effects in pathological conditions with pronounced inflammation, reducing the concentration of proinflammatory cytokines, cyclooxygenase‐2 (COX‐2), prostaglandin E2 (PGE_2_), NO, leukotrienes, and other inflammatory mediators (Ryu et al. 2017). Activation of p38 MAPK correlates with the production of various proinflammatory molecules, positioning it as a prospective therapeutic target for autoimmune conditions (Kim, Choe, and Lee 2022). SHH can also reduce p38 activation (Nyiramana et al. 2020). This mechanism of SHH is expected to contribute to reducing inflammation in RA.

Beyond these anti‐inflammatory mechanisms, how did SHH's role in immune cell regulation (activation of M1 macrophages, inhibition of M2 macrophage activity, and reduction of PD‐1 and PD‐L1 expression on TAM) contribute to the reduction of inflammation in the CIA model? At present, drawing a direct correlation remains challenging. Imbalances in the ratio of CD4+/CD8+ lymphocytes and M1/M2 macrophages within the synovium contribute to the development of RA (Canavan et al. 2021; Kurowska‐Stolarska and Alivernini 2022; Lowe et al. 2023). These imbalances may be influenced by therapies targeting PD‐1 and PD‐L1. Previous studies have shown that the histological level of PD‐1 correlated with the severity of synovial inflammation (Canavan et al. 2021; Raptopoulou et al. 2010). In the synovial tissue of RA patients, the expression of PD‐1 and PD‐L1 is notably increased. In addition, increased expression of PD‐1 is observed in CD4+ and CD8+ T cells within the synovial fluid of RA patients compared to their peripheral blood (Luo et al. 2018). This increase in specific T cells within the synovial tissue is associated with disease progression (Gordon et al. 2017; Lowe et al. 2023). Notably, there are significant differences in gene expression between PD‐1^+^ and PD‐1^−^ CD4+ cells circulating in these patients. A triple disease‐modifying antirheumatic drug has been shown to decrease the CD4+ PD‐1^+^ gene signature (Lowe et al. 2023). SHH treatment may influence the gene profile of CD4 + PD‐1^+^ cells and macrophage polarization through several pathways.

Additionally, in a phase 2 clinical trial (NCT02639065) for patients with advanced esophageal and gastroesophageal junction adenocarcinoma, the use of the PD‐L1 inhibitor Durvalumab resulted in a heightened presence of anti‐inflammatory macrophages (Mamdani et al. 2021). LPS treatment also increases M2 macrophage markers. The chronic inflammatory condition of RA is often linked to tissue dysfunction, leading to imbalances in physiological systems rather than being directly associated with host defense or tissue repair (Medzhitov 2008). We propose that SHH‐regulated PD‐1 and PD‐L1 expression could provide a potential pathway to alleviate inflammatory diseases like RA through its direct or indirect influence on inflammatory macrophage development.

So which bioactive compounds in SHH have anti‐inflammatory, anticancer, and immunomodulatory effects? Various bioactive substances in SHH, including glycosaminoglycans (Dhahri et al. 2020), terpenes (Ohyoshi, Zhao, and Kigoshi 2022; Petraki et al. 2015), and alkaloids (Kigoshi et al. 1990), exhibit multiple pharmacological effects. In previous studies, we identified glycosaminoglycans as the main constituents of SHH. The primary glycosaminoglycan was identified as heparan sulfate through techniques such as agarose gel electrophoresis and gel filtration chromatography on Superdex 200 HR, following established methodologies (Dietrich and Dietrich 1976; Yoon, Choi, and Choi 2010). Heparan sulfate plays a crucial role in immune regulation (Ryu et al. 2016). However, SHH's pharmacological superiority to pure heparan sulfate is notable. This may be due to the presence of other ingredients in SHH, which enhance its pharmacologic effects compared to the single ingredient.

SHH may have additional anti‐inflammatory properties that require further investigation. Detailed mechanistic analysis in future research is crucial to understanding these properties better. Considering these factors, SHH appears to be a promising candidate for further study. Developing effective strategies for managing irAEs in patients undergoing ICIs treatment is vital. These strategies are essential for enhancing treatment safety and tolerability while preserving the potential for durable antitumor responses. Furthermore, innovative interventions or agents that protect against irAEs during such therapies could revolutionize immuno‐oncology. This is particularly crucial for patients with autoimmune or interstitial lung diseases, who are typically excluded from such treatments.

## 5 Conclusions

SHH could be a promising I‐O agent, given its anticancer properties and potential to mitigate the unintended side effects of ICIs.

## Author Contributions

**Ji Hyeon Ryu:** formal analysis (equal), investigation (equal), methodology (equal), software (equal). **Min Seok Song:** visualization (equal), writing – review and editing (equal). **Marie Merci Nyiramana:** investigation (equal), methodology (equal). **Anjas Happy Prayoga:** visualization (equal). **Dang Long Cao:** visualization (equal). **Gyeong‐Won Lee:** writing – review and editing (equal). **Hyuk‐Kwon Kwon:** writing – review and editing (equal). **Dawon Kang:** conceptualization, methodology (equal), software (equal), validation, writing – original draft preparation, writing – review and editing (equal).

## Conflicts of Interest

The authors declare no conflicts of interest.

## Data Availability

The data associated with this study are not stored in a publicly accessible repository. However, the authors are willing to provide the data upon reasonable request. To access the data, please get in touch with Prof. Dawon Kang (dawon@gnu.ac.kr).
